# Giant Solitary Sinonasal Enchondroma: A Rare Case Report

**DOI:** 10.1007/s12070-024-04620-z

**Published:** 2024-04-13

**Authors:** Santiago Almanzo, Miguel Saro-Buendía, Sara Iommi Diez, Catalina Bancalari Díaz, Alfonso García Piñero, Miguel Armengot Carceller

**Affiliations:** 1 Servicio de Otorrinolaringología, Hospital Universitario y Politécnico La Fe, Avinguda Fernando Abril Martorell 106, Valencia, 46026 España; 2 Departament de Cirugia, Facultat de Medicina i Odontología, Universitat de València, Valencia, España; 3 Servicio de Medicina Familiar y Comunitaria, Hospital Universitario y Politécnico La Fe, Valencia, España

**Keywords:** Sinonasal tumor, Solitary enchondroma, Enchondromatosis, Secondary chondrosarcoma

## Abstract

Enchondromas, common benign intramedullary cartilaginous tumors, typically arise asymptomatically in metaphysis and diaphysis. We report a case diagnosed and treated for a giant sinonasal enchondroma, an extremely uncommon localization. Few cases have been reported in this location, and additional data is needed to draw conclusions regarding the potential for malignancy.

**Supplementary Information:**

The online version contains supplementary material available at 10.1007/s12070-024-04620-z.

## Introduction

Enchondromas are common, benign, and usually asymptomatic tumors forming hyaline cartilage, primarily in the metaphysis and diaphysis [1]. They are one of the most common osseous neoplasms, representing 12–24% of all benign bone tumors and 3–10% of all bone tumors [2]. Typically solitary and usually found in the small tubular bones of the hand, they may present as multiple lesions in conditions like Ollier’s disease (enchondromatosis) and Maffucci syndrome (enchondromatosis associated with hemangiomas). They frequently have a multinodular architecture, comprised by nodules of cartilage separated by bone marrow [1]. There’s a potential risk, up to 2%, of developing a secondary chondrosarcoma [3]. We present the case of a patient treated and diagnosed at our center, accompanied by a literature review due to the scarce prevalence in this location.

## Case Report

**Fig. 1:**
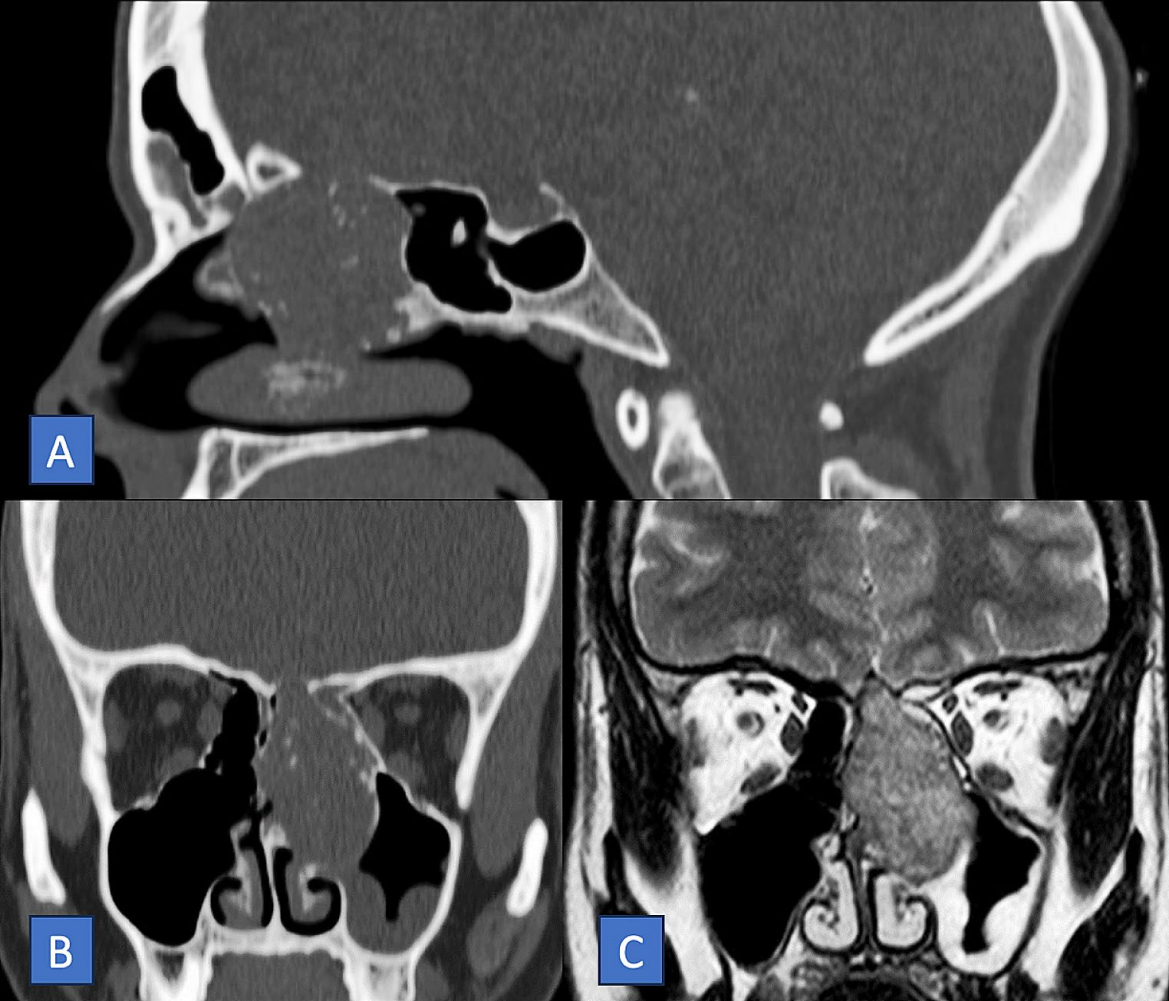
**A**: (Sagittal CT) A soft consistency tumor developing from the middle turbinate and occupying the ethmoid cells. **B**: (Coronal CT) Hypodense soft tissue mass in the ethmoidal area showing expansive growth obstructing the left frontal sinus. **C**: (Coronal T2-weighted MRI) high signal intensity mass with scattered microcalcifications within

**Fig. 2:**
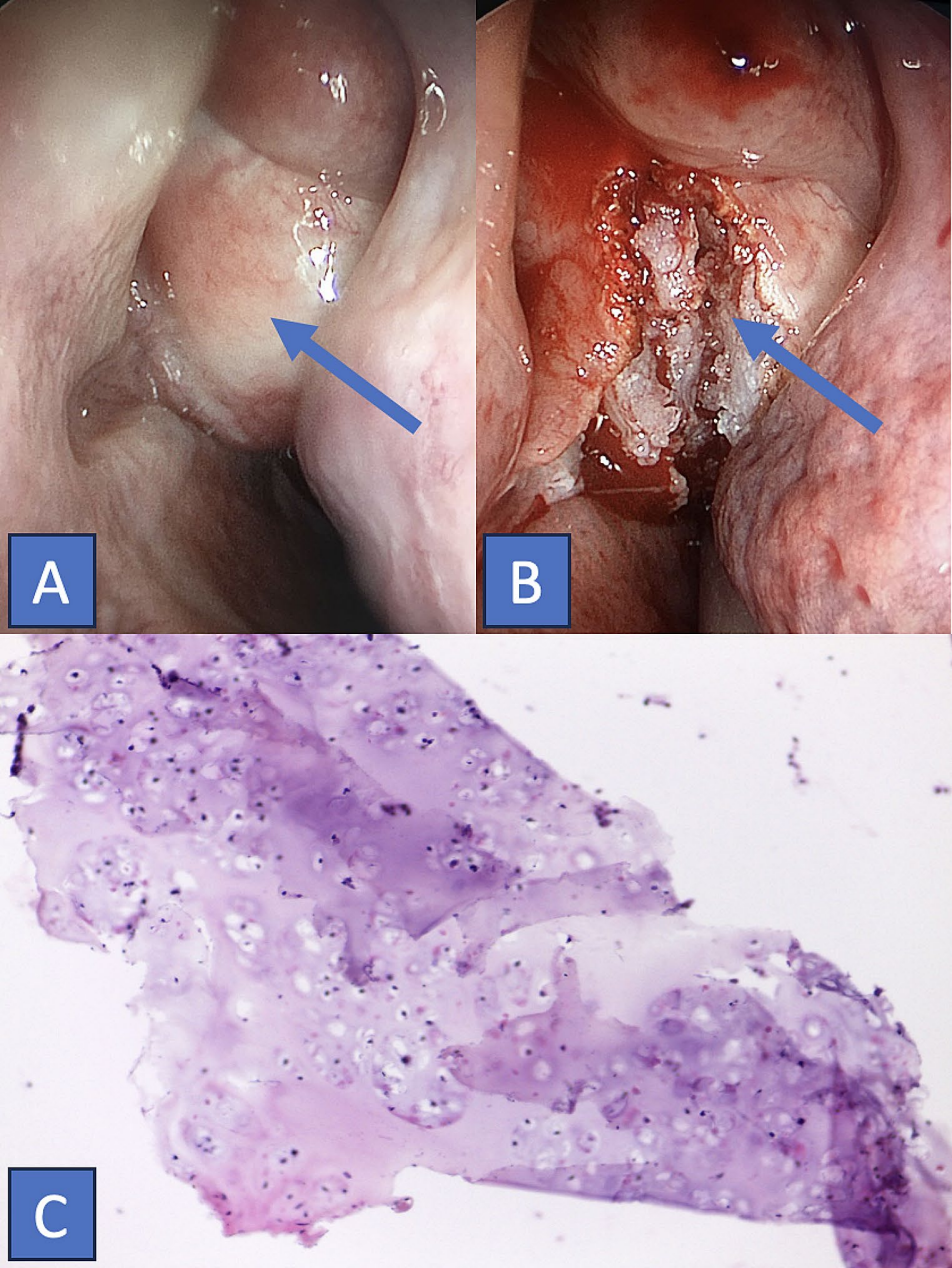
**A-B**: (Intraoperative findings) Tumor with a fibrocartilaginous appearance covered by normal mucosa (A). Upon tumor dissection (B), the presence of cartilaginous content becomes evident. **C**: (Microscopy) Hyaline cartilaginous tissue arranged in lobules with peripheral bony rim and fibrous tissue. Increased cellularity, with some chondrocytes exhibiting binucleation and mild pleomorphism. Foci of cortical rupture and osseous permeation present

A 32-year-old woman presented at our hospital with a four-month history of left nasal airway obstruction. Nasal endoscopy revealed a pale, firm, whitish mass originating from the inferior turbinate. Centered in the ethmoidal region, completely occupying the nasal cavity, and displacing the septum and turbinates. Computed tomography (CT) and magnetic resonance imaging (MRI) showed a sinonasal tumor involving the inferior turbinate, left maxillary sinus, and part of the ethmoid (Fig. 1). Through CT (Fig. 1A-B), the lesion was expansive, exhibited hypodensity, and appeared to be associated with the middle turbinate. It did not exhibit aggressive features but rather displayed well-defined, abrupt margins and chondral calcifications within its structure. Endoscopic surgery with the help of computer-assisted navigation was performed for the left nasal cavity, excising a fibrocartilaginous mass covered with normal mucosa (Fig. 2A-B). Macroscopic tumor-free margins were achieved: posteriorly to the sphenoid sinus, anteriorly to the frontal sinus, laterally to the orbit, and medially to the nasal septum. During the intervention, a minor dehiscent area was detected in the ethmoid roof, with a cerebrospinal fluid leak. The specific defect, measuring less than 5 mm, was situated in the left cribriform plate posterior to the crista galli. Repair involved the utilization of a free mucosal flap from the left nasal floor, complemented by tissue glue. Pathological analysis identified mostly enchondromatous features, such as hyaline cartilaginous tissue arranged in lobules with a peripheral bony rim and fibrous tissue, exhibiting increased cellularity and binucleation, with mild pleomorphism in some chondrocytes. Some focal points suggestive of low-grade chondrosarcoma were found (Fig. 2C). No mitosis or areas of necrosis were observed. Immunohistochemical techniques revealed expression of ERG. The patient recovered well. Based on the definitive histological diagnosis and literature review, clinical follow-ups included quarterly nasal endoscopy in the first year, followed by semiannual check-ups, along with annual MRI imaging. The patient has been recurrence-free for 5 years of follow-up.

## Discussion

Enchondromas are benign tumors originating from the intramedullary matrix of bones, composed of nodules of hyaline cartilage [4]. They are typically solitary and have a high prevalence in hand bones, but can occur in other locations and be multiple, as seen in Ollier’s disease and Maffucci syndrome [5, 6]. Solitary cases usually manifest between the ages of 20 and 40, with no gender prevalence, while multiple cases debut in childhood or adolescence [7]. They can be asymptomatic, or, as in our case due to its location, grow and cause nasal respiratory insufficiency, eventually leading to pain and fractures due to compression.

The diagnosis is established through a combination of clinical, radiological, and histopathological findings. CT and MRI are useful tools for assessing the extent and planning treatment, which will depend on the size and location. On CT, these lesions typically appear as multiple osteolytic lesions with oval, linear, and/or pyramidal shapes and well-defined margins in the metaphysis and/or diaphysis of long tubular bones and flat bones. On MRI, they present as lobulated lesions with intermediate signal intensity on T1-weighted images and predominantly high signal intensity on T2-weighted sequences [8].

The differential diagnosis with low-grade chondrosarcoma, both through imaging and histopathology, is not always straightforward. These tumors typically exhibit low cellularity, with small, uniform chondrocytes without atypia within an abundant matrix of hyaline cartilage and some calcifications [1]. In our case, cortical disruption raised uncertainty in the diagnosis, as such findings are common in low-grade chondrosarcomas [3].

Surgery is the primary treatment and can be curative in most cases. For enchondromas in other locations, recurrence is common if the excision is incomplete, making a surgical approach aimed at achieving clear margins the standard [9, 10]. Due to the potential for transformation into chondrosarcoma, long-term follow-up with nasal endoscopies and imaging tests is advisable.

## Conclusion

This is the second case published in the literature of solitary sinonasal enchondroma. The first case was reported in 2013 in a 10-year-old child, with only a 2-year follow-up [10]. We report this case to emphasize the rarity of this pathology, with only 2 cases published, including ours, and the lack of knowledge or guidelines regarding the treatment and follow-up for these patients. Therefore, it is crucial to report more similar cases to optimize the management of future cases.

### Electronic Supplementary Material

Below is the link to the electronic supplementary material.

Supplementary Material 1

## Abbreviations

CT: Computed tomography
MRI: Magnetic resonance imaging

## Declarations

The participant has consented to the submission of the case report to the journal and the publication of the images in Figs. 1 and 2.
