# Supplementary material for: Giant Solitary Sinonasal Enchondroma: A Rare Case Report
Source: Indian J Otolaryngol Head Neck Surg. 2024 Apr 13;76(4):3576–9. doi: 10.1007/s12070-024-04620-z (PMC11306484; doi:10.1007/s12070-024-04620-z)
Supplement: Supplementary file 1 — Supplementary Material 1 [file 12070_2024_4620_MOESM1_ESM.docx]

**Compliance with Ethical Standards**

Fundings and supports: None.

Conflict of Interest: The authors declare that they have no conflict of interest.

The participant has consented to the submission of the case report to the journal and the publication of the images in Figure 1 and Figure 2.

Ethical approval was waived by the local Ethics Committee of Hospital Universitario y Politécnico La Fe in view of the retrospective nature of the study and all the procedures being performed were part of the routine care.
